# Psychometric properties of the Arabic versions of the long (27 items) and short (13 items) forms of the interpersonal mindfulness scale (IMS)

**DOI:** 10.1186/s12888-024-05674-7

**Published:** 2024-04-03

**Authors:** Feten Fekih-Romdhane, Diana Malaeb, Vanessa Azzi, Rabih Hallit, Mariam Dabbous, Fouad Sakr, Sahar Obeid, Souheil Hallit

**Affiliations:** 1 The Tunisian Center of Early Intervention in Psychosis, Department of Psychiatry “Ibn Omrane”, Razi hospital, Manouba, 2010 Tunisia; 2 Faculty of Medicine of Tunis, Tunis El Manar University, Tunis, Tunisia; 3 College of Pharmacy, Gulf Medical University, Ajman, United Arab Emirates; 4 School of Medicine and Medical Sciences, Holy Spirit University of Kaslik, P.O. Box 446, Jounieh, Lebanon; 5 Department of Infectious Disease, Bellevue Medical Center, Mansourieh, Lebanon; 6 Department of Infectious Disease, Notre Dame des Secours, University Hospital Center, Byblos, Lebanon; 7 School of Pharmacy, Lebanese International University, Beirut, Lebanon; 8 École Doctorale Sciences de la Vie et de la Santé, Université Paris-Est Créteil, Institut Mondor de Recherche Biomédicale, Créteil cedex, France; 9 Social and Education Sciences Department, School of Arts and Sciences, Lebanese American University, Jbeil, Lebanon; 10 Psychology Department, College of Humanities, Effat University, Jeddah, 21478 Saudi Arabia; 11 Applied Science Research Center, Applied Science Private University, Amman, Jordan

**Keywords:** Interpersonal mindfulness, Psychometrics, Test adaptation, Lebanon, Arabic

## Abstract

**Background:**

There is a lack of measures and data on interpersonal mindfulness from non-Western cultures, which can hinder advances in our understanding of the construct, its conceptual representation, and its effects on human connection and relationships within different cultural settings. To fill this gap and help spark future research in this area in the Arab world, the current study aimed to examine the psychometric properties of an Arabic translation of the 27-item and the 13-item versions of the interpersonal Mindfulness Scale (IMS) in a sample of Arabic-speaking adolescents from the general population.

**Methods:**

A web-based survey was conducted in a sample of 527 Lebanese community adolescents (Mean age = 15.73 ± 1.81 years; 56% females). The IMS was translated from English into Arabic using the forward-backward translation method. Participants completed the long and short forms of the IMS, as well as the Buss–Perry Aggression Questionnaire-Short Form (BPAQ-SF), and the 5-item Brief Irritability Test.

**Results:**

Confirmatory factor analyses provided support to the four-factor structure of both the 27-item and the 13-item IMS (i.e., Presence, Awareness of Self and Others, Nonjudgmental Acceptance, and Nonreactivity). The original and the short form versions of the IMS yielded excellent internal consistency in our sample, with a Cronbach’s α coefficients of 0.95 and 0.90, and McDonald’s omega coefficients of 0.95 and 0.90, respectively. Multigroup comparisons suggested the factorial invariance of the Arabic 27-item and 13-item IMS between male and female participants at the metric, configural, and scalar levels. Finally, the concurrent validity of both full-length and short form of the IMS appeared to be good and comparable, as attested by patterns of correlations in expected directions with outcome variables (i.e., aggression, anger, hostility, and irritability).

**Conclusion:**

The present findings provide support for the good psychometric qualities of the Arabic translation of the IMS in both long and short forms, suggesting that these scales are suitable for use to measure interpersonal mindfulness in Arabic-speaking youth, at least in Lebanon. We expect that the IMS, in particular its shortest form, will prompt more systematic investigation of interpersonal mindfulness in the Arabic-speaking populations, especially with regard to enhancing healthy communications with others and building effective social relationships.

## Introduction

The concept of mindfulness, mainly as a self-reported trait, has triggered a rapidly expanding research interest over the past years [1]. Mindfulness refers to the tendency of paying attention to, and being aware of, the present moment in a nonjudgmental and nonreactive way [2, 3]. A strong evidence exists to support that trait mindfulness may positively influence several life’s areas. The general disposition to be mindful was found to be closely related to an array of positive psychological outcomes, including better emotion regulation [4], lower levels of psychological distress (e.g., depression, anxiety, stress [5], burnout [6], social anxiety [7]), high self-efficacy, increased well-being [8], more healthy [9] and prosocial [10] behaviors, as well as better quality of interpersonal relationships (i.e., greater marital quality [11–13], relationship satisfaction [14, 15], and relationship functioning [16]). In particular, previous studies indicated that mindfulness is associated with a number of interpersonal skills, including active listening [17], empathetic communication [18], and establishing more stable and satisfied romantic relationships [19].

Research attention on mindfulness has initially and mostly focused on the individual, and has for a long time regarded the concept as a solitary, internal practice. In contrast, very little knowledge exists on how mindfulness manifests during the multiple exchanges in daily interpersonal interactions [20]. The construct of interpersonal mindfulness is distinct from the general construct of (trait) mindfulness. Being interpersonally mindful implies to remain attentive to another person, and not only to oneself. It may, therefore, involve attributes and skills that are not manifested in general trait mindfulness. Besides, interpersonal mindfulness reflects the multitude of experiences that can directly shape the quality of one’s interpersonal interactions and, in turn, the quality (processes and outcomes) of their relationships with other people [20, 21]. Examples of mindfulness occurring within the interpersonal context include giving and maintaining undivided attention to the speaker while being non-judgmental and focusing on the present inner experiences, or being responsive, thoughtfully reactive, during conversations [20]. As a result, the other is likely to feel understood and important [22]. Hence, interpersonal mindfulness may enhance the quality of awareness to, and sensing of, more subtle cues and nonverbal communication (e.g., verbal tone, apparent mood, and body language), thereby fostering attunement to other’s feelings and thoughts [23]. In this regard, prior research showed that interpersonal mindfulness is closely linked to emotional intelligence, authenticity, empathy, and active-empathic listening, effective communication, and has, in turn, many positive implications for social relationships and functioning (e.g., feelings of social connectedness, increased friendship quality, relationship satisfaction) [20, 21, 24, 25]. In addition, a person who is interpersonally mindful is likely to exhibit skillfully responses to strong emotions arising during interpersonal conflicts, such as contempt or anger [11]. There is also strong evidence to suggest that mindfulness may be effective in reducing aggression and violence [26], and improving irritability [27]. All these interpersonal and social outcomes are determinant for maintaining a good overall health and well-being [28].

Overall, the concepts of mindfulness and interpersonal mindfulness reflect different characteristics [20, 21, 29–32], and appear to involve different constructs [20, 30]. Although a growing amount of research has investigated the effects of mindfulness on interpersonal relationships (e.g., parent-child [33], teacher-student [31], friendship [21], and marital [34] relationships), and despite evidence on the value of considering mindfulness in social contexts, research was slow to develop and apply measures that specifically capture the construct of interpersonal mindfulness. Yet, there are very few valid and reliable measurement instruments that specifically cater for interpersonal mindfulness [35]. In the past two decades, researchers have attempted to develop or adapt measures to assess aspects of mindfulness relevant to interpersonal interactions within specific situations and social roles, such as the Interpersonal Mindfulness in Parenting Scale [30], the Mindfulness in Teaching Scale [31], and the (Romantic) Relationship Mindfulness Measure [36]. Given the wide range of social interactions in which individuals may engage, Pratscher et al. [20] were the first to create and validate a non-context-specific measure that can be more useful in broader interpersonal contexts, i.e. the Interpersonal Mindfulness Scale (IMS).

The IMS was designed to evaluate the extent to which one can be mindful when listening or speaking to another person, by measuring the quality of mindfulness specific to interpersonal interactions [20, 21]. The IMS is composed of 27 items, which were selected after a process including item generation, item deletion, and psychometric evaluation across five independent samples [20]. The scale measures a high-order factor of interpersonal mindfulness, with the following four sub-factors: (1) Presence, which includes being in the present moment (e.g., paying attention while listening) during interpersonal interactions; (2) Awareness of Self and Others, which is centered around being aware of internal personal experiences (e.g., moods, emotions, bodily sensations) as well as intentions, moods, and nonverbal cues of others during an interpersonal interaction; (3) Nonjudgmental Acceptance, which refers to listening nonjudgmentally and accepting experiences as they are; and (4) Non-reactivity, which reflects taking time before responding during an interaction. All four subscales and total scale showed good internal consistency, test–retest reliability, and construct validity [20].

For an easier, faster and less burdening administration of the scale, as well as an increased feasibility in future research and clinical applications, Pratscher et al. [37] developed two shortened versions, a 17-item and a 13-item IMS-short forms (IMS-SF), while maintaining the four-factor structure of the original scale. The shortest version of the IMS-SF (IMS-SF-13) demonstrated strong psychometric properties in terms of reliability and validity of the construct, and was recommended by the developers for use for most research (e.g., associations with other variables or examining group-level changes) [37]. The 27-item IMS has recently been translated and adapted to the Persian language among Iranian undergraduate students [38]. Aside from this validation study, there exists little psychometric information on the IMS full-length and short forms in other cultures and countries, thereby limiting knowledge on the interpersonal and relational effects of mindfulness.

As for Arabic countries, for example, very few studies have focused on positive psychology in general, and mindfulness in particular [39]. It is only very recently that two scales were translated and validated in the Arabic language to assess the mindfulness construct broadly (i.e., the Freiburg Mindfulness Inventory [40], the Cognitive-Affective Mindfulness Scale [41]). However, no Arabic scales are available to date to measure the specific construct of interpersonal mindfulness in Arabic-speaking populations. Although prior research among different Arabic-speaking community people around the globe [39, 42–45] revealed that mindfulness is effective in improving psychological wellbeing and compatible with the Arab cultural and religious background, the conceptualization of mindfulness may not be as universal as is commonly assumed [46]. Indeed, earlier experiences demonstrated that cultural and religious factors are important to consider when dealing with mindfulness, and that previously tested mindfulness-based psychological interventions needed to be tailored to fit aspects of Arab culture (e.g., [47]). Thus, the lack of measures and data from non-Western cultures can hinder advances in our understanding of interpersonal mindfulness, its conceptual representation and its effects on human connection and relationships within different cultural settings. In addition, making sound measures of mindfulness available in the Arabic language might help in developing effective mindfulness-based psychological interventions that are culturally congruent to the needs of people from Arab cultural backgrounds. Such interventions can offer a complementary but essential role alongside traditional interventions in preventing and/or treating psychological issues in Arab environments, where the burden of mental disorders is steadily on the rise [48], and stigma around mental health issues is still highly prevalent [49–51]. In particular, adolescents’ mental health problems in the MENA, a region that comprises the highest proportion of adolescents worldwide, were considered by some researchers as “a silent Epidemic” [52]. Therefore, the validation and application of the IMS among Arab adolescents is highly relevant to clinical practice, especially since adolescence represents a critical period of development and a window of opportunity, where brain changes can be highly vulnerable to environmental influences, such as mindfulness training [53]. To fill all these gaps and help spark future research in this area in the Arab world, the objective of this study was to examine the psychometric properties of an Arabic translation of the 27-item and the 13-item versions of the IMS in terms of structural validity, reliability, cross-sex invariance and concurrent validity among adolescents from the general population of Lebanon. Consistent with the previous psychometric studies using other linguistic versions of the 27-item and the 13-item versions of the IMS (i.e., English [20, 37], Persian [38]) which provided support for a four-factor structure, adequate reliability and validity, it is hypothesized that confirmatory factor analysis will replicate this factor structure, and that both versions of the IMS will show good internal consistency and concurrent validity in our sample of Arabic-speaking adolescents.

## Methods

### Participants and procedures

A total of 527 adolescents completed the survey (mean age: 15.73 ± 1.81; 56% females). A convenient sampling method (snowball technique) was used to collect data during April-May 2023. After completing a training with the research team, five university students were asked to collect data via a Google Form link; they were asked to forward the link to people they know, who in turn were asked to forward the link to other family members and friends. Inclusion criteria for participation included being of a resident and citizen of Lebanon and aged between 12 and 18 years. Excluded were those who refused to fill out the questionnaire. Internet protocol (IP) addresses were examined to ensure that no participant took the survey more than once. Participants were asked in the introductory paragraph to take their parents’ consent before filling the survey. After providing digital informed consent, participants were asked to complete the instruments described above, which were presented in a pre-randomized order to control for order effects. The survey was anonymous and participants completed the survey voluntarily and without remuneration [65].

### Translation Procedure

The forward-backward translation approach was used for the interpersonal mindfulness scale. The English version was translated to Arabic by a Lebanese certified translator (with more than 10 years of experience) who was completely unrelated to the study. Afterwards, a Lebanese psychologist (a university teacher and a PhD holder) with a full working proficiency in English, translated the Arabic version back to English. The translation team ensured that any literal and/or specific translation was balanced. The initial and translated English versions were compared to detect/eliminate any inconsistencies and guarantee the accuracy of the translation by a committee of experts composed of the research team, one psychologist, one psychiatrist and the two translators [54]. An adaptation of the measure to the Arab context was performed, and sought to determine any misunderstanding of the items wording as well as the ease of items interpretation; therefore, ensure the conceptual equivalence of the original and Arabic scales in both contexts [55]. After the translation and adaptation of the scale, a pilot study was done on 30 participants to ensure all questions were well understood; no changes were applied after the pilot study.

### Minimum sample size

According to Mundfrom et al. [56], the minimum sample size required to have enough statistical power to conduct a confirmatory factor analysis (CFA) should be 3–20 times the number of items in the scale, which was attained in our sample; by collecting data from a number of participants that exceeds the minimum sample size will give more statistical power to the study, resulting in more accurate and reliable results. At the end of the data collection, 527 Lebanese community adolescents completed the survey (Mean age = 15.73 ± 1.81 years [min = 12; max = 18]; 56% females).

### Measures

#### Demographics

Participants were asked to provide their demographic details consisting of age and sex.

#### The interpersonal mindfulness scale (IMS)

The 27-item IMS [20] and the IMS-SF-13 [37] are developed by Pratscher and collaborators in 2019 and 2022, respectively. They evaluate interpersonal mindfulness through four sub-scales: Presence (e.g., “rather than being distracted, it is easy for me to be in the present moment while I am interacting with another person”), Awareness of self and others (e.g., “when I am with other people, I am aware of my moods and emotions”), Non-judgemental acceptance (e.g., “when in discussion, I accept others have opinions different from mine”), and Non-reactivity (e.g., “when I receive an angry text/email from someone, I try to understand their situation before responding”). Both measures are based on a 5-point Likert scale from 1 (almost never) to 5 (almost always). Scores range from 13 to 65 for the IMS-SF-13 and from 27 to 135 for the 27-item IMS, with greater scores indicating higher level (greater presence) of interpersonal mindfulness [20, 37].

#### The Buss–Perry aggression questionnaire-short form (BPAQ-SF)

The BPAQ-SF is a short version of the BPAQ developed by Bryant and Smith in 2001 [57], and it contains 12 items divided into four subscales and rated on a 5-point Likert scale. In the original validation, reliability coefficients of the subscales were the following: 0.78 for physical aggression, 0.72 for verbal aggression, 0.83 for anger, and 0.84 for hostility [57]. The Cronbach’s alpha values of the Arabic validated version used in the present study [58] were as follows: physical aggression (α = 0.66), verbal aggression (α = 0.55), hostility (α = 0.72), and anger (α = 0.71). In the present sample, subscales yielded the following internal consistency reliability coefficients: physical aggression (ω = 0.78 and α = 0.78), verbal aggression (ω = 0.66 and α = 0.63), anger (ω = 0.77 and α = 0.76), and hostility (ω = 0.78 and α = 0.78). Total scores range from 12 to 60, with higher scores indicating higher levels of aggression.

#### The brief irritability test

The BITe was developd by Holtzman et al. in 2015 [59]. It is a five-item self-report tool that assesses irritability as a state over the last two weeks [59]. Each item is rated on a 6-point Likert scale ranging from 1 (never) to 6 (always). A total score is calculated by summing the score of each item. Total scores range from 5 to 30, with higher scores reflecting more irritability. The original version showed excellent internal consistency (α = 0.88). The Arabic validated version of the scale was used [60], which has also shown excellent reliability (Cronbach’s alpha and McDonald’s omega coefficient values were of 0.88). In the present sample, internal consistency coefficients values were of ω = 0.88 and α = 0.88.

### Analytic strategy

#### Confirmatory factor analysis

There were no missing responses in the dataset.

Since the structure of the scale is known, we used data from the total sample to conduct a CFA using the SPSS AMOS v.29 software. Our intention was to test the four-factor model obtained from the original study [20]. The normed model chi-square (χ²/df), the Steiger-Lind root mean square error of approximation (RMSEA), the Tucker-Lewis Index (TLI) and the comparative fit index (CFI). Values ≤ 5 for χ²/df, and ≤ 0.08 for RMSEA, and 0.90 for CFI and TLI indicate good fit of the model to the data [61]. The absence of multicollinearity was verified through tolerance values > 0.2 and variance inflation factor (VIF) values < 5. Multivariate normality was not verified at first; therefore we performed non-parametric bootstrapping procedure (available in AMOS).

#### Sex invariance

To examine sex invariance of IMS scores, we conducted multi-group CFA [62] using the total sample. Measurement invariance was assessed at the configural, metric, and scalar levels [63]. Configural invariance implies that the latent scales variable(s) and the pattern of loadings of the latent variable(s) on indicators are similar across gender (i.e., the unconstrained latent model should fit the data well in both groups). Metric invariance implies that the magnitude of the loadings is similar across gender; this is tested by comparing two nested models consisting of a baseline model and an invariance model. Scalar invariance implies that both the item loadings and item intercepts are similar across gender and is examined using the same nested-model comparison strategy as with metric invariance [64]. Measurement invariance was determined if ΔCFI ≤ 0.010 and ΔRMSEA ≤ 0.015 or ΔSRMR ≤ 0.010 [62]. If invariant, we aimed to check for a difference in NS scores in terms of sex using the Student *t*-test.

#### Reliability and validity analyses

Composite reliability in both subsamples was assessed using McDonald’s ω and Cronbach’s alpha [65], with values greater than 0.70 reflecting adequate composite reliability. The total IMS-27 and IMS-13 scores followed a normal distribution, with skewness and kurtosis values varying between − 1 and + 1 [66]. To assess convergent and concurrent validity, we examined bivariate correlations between NS scores and the other scales included in the survey using the Pearson test. Based on Cohen [67], values ∼ 0.10 were considered weak, ∼ 0.30 were considered moderate, and ∼ 0.50 were considered strong correlations.

## Results

### Confirmatory factor analysis of different models

**Fig. 1:**
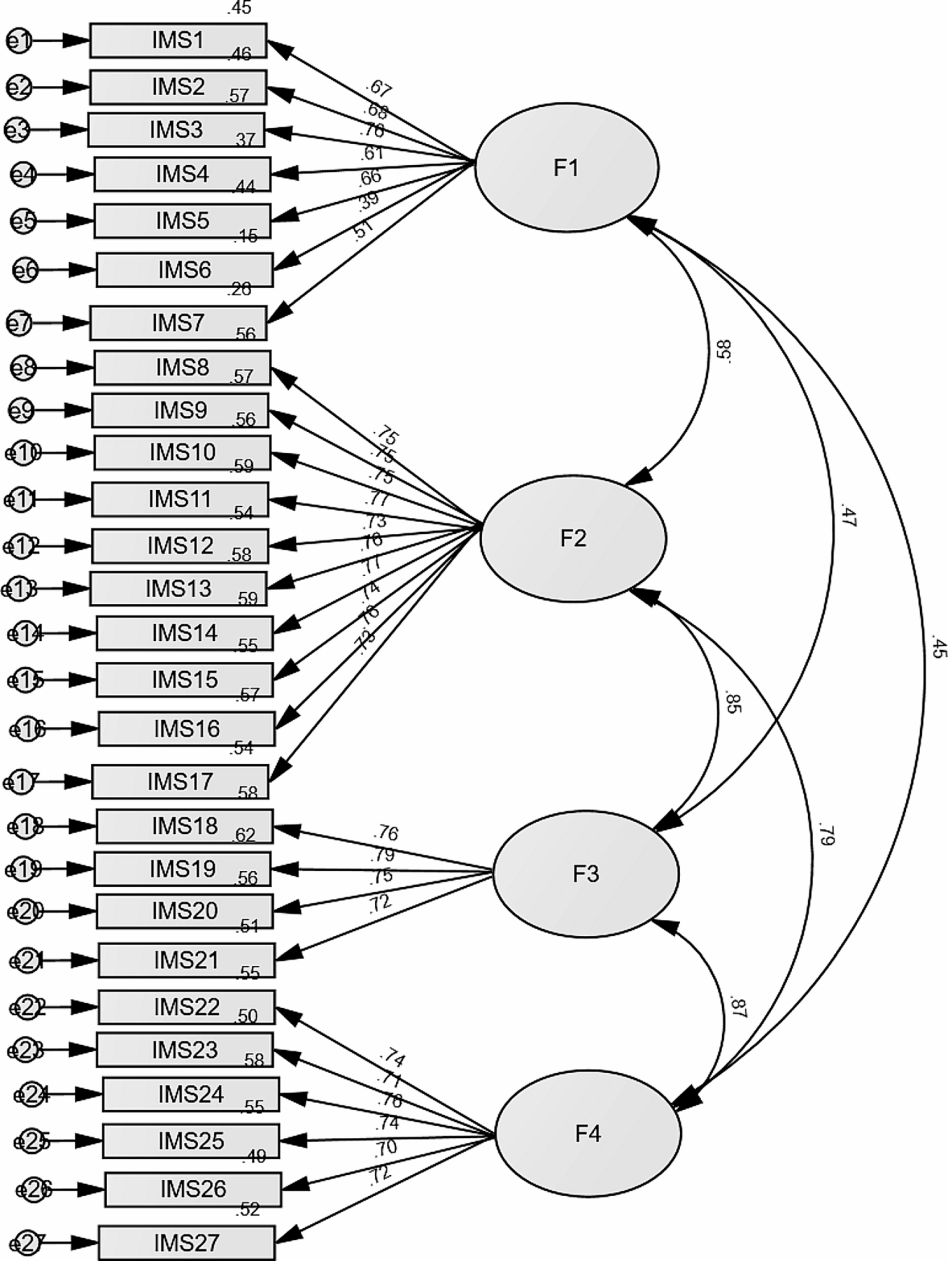
Standardized estimates of factor loadings of the interpersonal mindfulness scale − 27 items from the confirmatory factor analysis in the total sample

A CFA was conducted on the total sample according to the original 4-factor scale; the fit indices were acceptable as follows: χ^2^/df = 1117.72/318 = 3.52, *p* < .001, RMSEA = 0.069 (90% CI 0.065, 0.074), SRMR = 0.080, CFI = 0.899, and TLI = 0.889. The same was applied to the 13-item version of the scale; the fit indices were excellent as follows: χ^2^/df = 136.37/59 = 2.31, *p* < .001, RMSEA = 0.050 (90% CI 0.039, 0.061), SRMR = 0.036, CFI = 0.974, and TLI = 0.965. The standardized estimates of factor loadings for both models were all adequate (Table 1; Fig. 1). The reliability analysis for both scales were excellent as follows: 27-item IMS-27 (ω = 0.95 and α = 0.95), Presence (ω = 0.80 and α = 0.80), Awareness of self and others (ω = 0.93 and α = 0.93), Non-judgmental Acceptance (ω = 0.84 and α = 0.84), and Non-reactivity(ω = 0.87 and α = 0.87) and IMS-SF-13 (ω = 0.90 and α = 0.90), Presence (ω = 0.74 and α = 0.74), Awareness of self and others (ω = 0.85 and α = 0.85), Non-judgmental Acceptance (ω = 0.81 and α = 0.81), and Non-reactivity(ω = 0.74 and α = 0.74) respectively.

It is noteworthy that the fit indices of the one-factor structure of the IMS-27 (χ^2^/df = 1989.02/324 = 6.14, *p* < .001, RMSEA = 0.099 (90% CI 0.095, 0.103), SRMR = 0.077, CFI = 0.790, and TLI = 0.772) and IMS-13 (χ^2^/df = 550.42/65 = 8.47, *p* < .001, RMSEA = 0.119 (90% CI 0.110, 0.128), SRMR = 0.081, CFI = 0.835, and TLI = 0.802) showed poor results.

**Table 1:** Items of the interpersonal mindfulness scale (27-item IMS and IMS-SF-13) in english and standardized estimates of factor foadings from the confirmatory factor analysis in the total sample

Items	The 27–item IMS	The IMS-SF-13	Mean ± SD
**Presence (Factor 1)**			
1. When I am conversing with another person, I am fully engaged in the conversation.	0.67		3.34 ± 1.05
2. When a person is talking to me, I find myself thinking about other things, rather than giving them my full attention.	0.68	0.74	3.33 ± 1.01
3. I find myself listening to someone with one ear while doing something else at the same time.	0.76	0.74	3.43 ± 1.00
4. When interacting with someone I know, I am often on autopilot, not really paying attention to what is actually happening in the moment.	0.61	0.62	3.30 ± 1.07
5. When I am with others, I am easily distracted and my mind tends to wander.	0.66		3.49 ± 1.08
6. I give the appearance of listening to another person when I am not really listening.	0.39		3.13 ± 1.06
7. Rather than being distracted, it is easy for me to be in the present moment while I am interacting with another person.	0.51		3.30 ± 1.01
**Awareness of self and others (Factor 2)**			
8. When I am with other people, I am aware of my moods and emotions.	0.75	0.74	2.98 ± 1.15
9. I listen for the meaning behind another person’s words through their gestures and facial expressions.	0.76	0.81	2.90 ± 1.15
10. I am aware of others moods and tone of voice while I am listening to them.	0.75		2.98 ± 1.11
11. I am aware of my facial and body expressions when interacting with others.	0.77	0.78	2.98 ± 1.12
12. When interacting with others, I am aware of their facial and body expressions.	0.73		2.97 ± 1.14
13. I pick up on the intentions behind what another person is trying to say.	0.76		3.00 ± 1.15
14. When I am interacting with another person, I get a sense of how they are feeling.	0.77		2.83 ± 1.15
15. I accept that another person’s current situation or mood might influence their behavior.	0.74		3.04 ± 1.11
16. When speaking to another person, I am aware of how I feel inside.	0.76		2.94 ± 1.12
17. I notice how my mood affects how I act towards others.	0.73	0.73	2.88 ± 1.13
**Non-judgmental Acceptance (Factor 3)**			
18. When interacting with others, I am aware of their facial and body expressions.	0.76	0.77	2.87 ± 1.16
19. I pick up on the intentions behind what another person is trying to say.	0.79	0.78	2.96 ± 1.13
20. I listen to another person without judging or criticizing them.	0.75	0.73	2.91 ± 1.13
21. I give the appearance of listening to another person when I am not really listening.	0.72		3.02 ± 1.12
**Non-reactivity (Factor 4)**			
22. In tense moments with another person, I am aware of my feelings but do not get taken over by them.	0.74		2.93 ± 1.16
23. When I receive an angry text/email from someone, I try to understand their situation before responding.	0.71	0.67	3.06 ± 1.12
24. When I am upset with someone, I notice how I am feeling before responding.	0.77		3.07 ± 1.10
25. I take time to form my thoughts before speaking.	0.74	0.72	3.11 ± 1.09
26. I think about the impact my words may have on another person before I speak.	0.70	0.71	3.15 ± 1.08
27. Before I speak, I am aware of the intentions behind what I am trying to say.	0.72		2.95 ± 1.12

### Sex invariance

Indices in Table 2 indicate that configural, metric, and scalar invariance was supported across sex in the total sample for the 27-item IMS and the IMS-SF-13. No significant difference was found between females and males in terms of the 27- and 13-item IMS scores (Table 3).

**Table 2:** Measurement invariance across sex

Model	χ²	df	CFI	RMSEA	SRMR	Model Comparison	Δχ²	ΔCFI	ΔRMSEA	ΔSRMR	Δdf	p
Model 1: IMS												
Configural	1782.95	636	0.862	0.059	0.080							
Metric	1818.78	659	0.860	0.058	0.096	Configural vs. metric	35.83	0.002	0.001	0.016	23	0.042
Scalar	1836.47	680	0.861	0.057	0.097	Metric vs. scalar	17.69	0.001	0.001	0.001	21	0.668
Model 2: IMS-SF-13												
Configural	250.60	118	0.956	0.046	0.051							
Metric	256.12	127	0.957	0.044	0.053	Configural vs. metric	5.52	0.001	0.002	0.002	9	0.786
Scalar	258.56	136	0.959	0.041	0.053	Metric vs. scalar	2.44	0.002	0.003	< 0.001	9	0.982

*Note* CFI = Comparative fit index; RMSEA = Steiger-Lind root mean square error of approximation; SRMR = Standardised root mean square residual

**Table 3:** Comparison of interpersonal mindfulness scale (IMS-27) and subscales scores between sexes

	IMS-27 total	Presence	Awareness of self and others	Non-judgmental Acceptance	Non-reactivity	IMS-13 total	
**Males**	82.98 ± 12.01	22.38 ± 3.68	30.03 ± 3.86	11.92 ± 2.22	18.66 ± 4.68	40.58 ± 7.63	
**Females**	81.43 ± 12.75	22.33 ± 3.73	29.58 ± 4.18	11.55 ± 2.41	17.97 ± 5.59	39.72 ± 8.32	
***t***	1.424	0.132	1.260	1.844	1.533	1.225	
***df***	525	525	525	525	525	525	
***p***	0.155	0.895	0.208	0.066	0.126	0.221	

Numbers in bold indicate significant *p* values

### Concurrent validity

IMS scores were positively and strongly correlated with IMS-SF-13 scores (*r* = .96, *p* < .001). In addition, higher IMS total scores were significantly and negatively correlated with physical aggression (*r* = − .13, *p* = .002), verbal aggression (*r* = − .15, *p* < .001), anger (*r* = − .20, *p* < .001), hostility (*r* = − .26, *p* < .001) and irritability (*r* = − .32, *p* < .001). Same correlations were found between IMS-SF-13 and physical aggression (*r* = − .14, *p* = .001), verbal aggression (*r* = − .18, *p* < .001), anger (*r* = − .21, *p* <. 001), hostility (*r* = − .27, *p* < .001) and irritability (*r* = − .33, *p* < .001). All correlations were small to moderate in magnitude.

## Discussion

The presence of mindfulness while interacting with others has proven to have numerous benefits, contributing to more positive and functional relationships [20, 21, 30, 32, 33]. Measures that specifically capture the interpersonal mindfulness construct are thus crucial for a better understanding of human behavior during dyadic interactions, and ways to improve relationship outcomes. The goal of the present study was to translate into Arabic and validate two scales that are specifically pertinent to mindfulness within the context of interpersonal interactions, i.e. the IMS and the IMS-SF-13. Findings showed that the two measures had a four-factor structure, excellent reliability coefficients, good concurrent validity (based on correlations with measures of aggression, anger, hostility, and irritability), and were measurement invariant across sex groups. These results suggest that the Arabic IMS and the IMS-SF-13 are suitable for use among Arabic-speaking adolescents, and may hopefully contribute to foster research on interpersonal mindfulness in Arab contexts. As the IMS-SF-13 showed good psychometric qualities while requiring fewer items, it should be a desirable alternative for future research as suggested by Pratscher et al. [37].

CFA provided additional support to the four-factor structure of the IMS (i.e., Presence, Awareness of Self and Others, Nonjudgmental Acceptance, and Nonreactivity), thus suggesting that mindfulness is likely multidimensional. These four dimensions may be relevant, allowing to obtain useful information about the processes implicated in interpersonal mindfulness training interventions [68], as well as a better understanding of the construct [11, 69]. At the same time, however, there are theoretical reasons to suggest that dimensions of mindfulness should be integrated into one single dimension [70]. Pratscher et al. [20] found a hierarchical solution in which four first-order factors loaded onto a single higher-order interpersonal mindfulness factor. Similarly, findings regarding the IMS-SF-13 supported a higher-order latent structure that can easily be interpreted with a single global score [37]. Accordingly, they recommended either examining each of the four subscales separately, or summing all items to derive a single assessment reflecting the continuum of interpersonal mindfulness levels [20]. It is of note that the major strength of the IMS is that it was initially conceptualized by Pratscher et al. [21] to be applicable to all types of interpersonal interactions. At the same time however, the measure tends to focus on mental and metacognitive skills and does not emphasize the role of the body, despite the body being central to the experience of mindfulness [71] and having a crucial role in attunement to other persons’ body language during mindful interpersonal interactions [21]. In future, researchers might consider adding items to the IMS that specifically focus on the role of the body in the interpersonal dynamic. Despite Lebanon being grappled with a multitude of crises that pose significant threats to mental wellbeing of its population (Beirut port explosion on August 4, 2020, severe economic crisis with a staggering economic inflation rate of 154.8% and a 75% reduction in income [72]), we believe that the Arabic version (translated into the read and written language) can be used in other Arab countries, where youth have been confronted with substantial challenges too, as evidenced by the increasing prevalence of psychological distress [73], limited opportunities, illegal migration [74], unemployment and a high rate of school dropout [75].

In our sample, the original and the short form versions of the IMS yielded excellent internal consistency, with a Cronbach’s α coefficients of 0.95 and 0.89, and McDonald’s omega coefficients of 0.95 and 0.90, respectively. These findings are consistent with those of the original validation studies [20, 37], and further corroborate the reliability of the measure. The Persian version of the 27-item IMS also revealed a scale with four subscales, with appropriate internal consistency [38]. As the IMS was originally developed in an American Western culture and context, and as the Arab and American cultures are viewed by scholars as cultural opposites [76], it was important to test whether its factor structure applies to individuals who grew up in an Arab cultural context. Indeed, compared to western American culture, interpersonal communication in the Arab culture is marked by several specific characteristics, including an emphasis on form over function, affect over accuracy, and image over meaning [76]. Interpersonal communication patterns tend, for example, to be ambiguous, indirect, emotionally rich and people-oriented in Arabs, as opposed to clear, direct, objective and object-oriented communication in Americans [76]. In addition, relationships are highly valued in Arab societies, where showing interest and taking time with people for extensive greetings (asking about each other’s health and families) is the norm, while American norms of simply waving in passing would be seen as rude. Arab people tend to share their emotions and life difficulties with others, and support each other during the good and the bad times. Therefore, paying attention and being, for example, “fully engaged in the conversation” are highly expected patterns of communication between Arabs. The finding that the original factor structure of the IMS is supported in our Lebanese Arabic-speaking sample suggests that cultural differences have not caused the interpersonal mindfulness construct to be assessed differently compared to the original US English-speaking sample. This can be explained by the fact that mindfulness is universal and non-culturally-dependent in nature, and seems to be free from environmental influences [77]. To confirm these assumptions, future research in other cultural backgrounds is still needed to confirm whether the four-factor model of interpersonal mindfulness holds up cross culturally.

Multigroup comparisons suggested the factorial invariance of the Arabic 27-item and 13-item IMS between male and female participants at the metric, configural, and scalar levels. Evidence for invariance across sex of the IMS has also been demonstrated in US non-clinical adults [37]. This supports that items are interpreted and understood in a same way across sex, and that the IMS functions nearly identically within male and female groups. Our study represents a first effort to provide data on the measurement invariance of the IMS across sex. Such evidence provides empirical support to enable researchers and psychotherapists using these Arabic versions of the IMS to interpret between-sex comparisons as true differences in interpersonal mindfulness levels, rather than a measurement artifact.

The 27-item and 13-item versions of the IMS demonstrated high correlations with one another, highlighting that the shortest form of the IMS retains equivalent measurement of the interpersonal mindfulness construct as suggested by Pratscher et al. [37]. In addition, the concurrent validity of both full-length and short form of the IMS appeared to be comparable, as attested by patterns of correlations in expected directions with outcome variables (i.e., aggression, anger, hostility, and irritability). The significant positive associations between mindfulness and lower aggression and irritability levels is consistent with previous findings [26, 27]. These results suggest that the IMS may be a valuable and relevant measure to enhance our understanding of the ways in which mindfulness relates to intrapersonal and interpersonal outcomes, and may be relevant to interpersonal interactions. As the construct of interpersonal mindfulness has only been conceptualized and the IMS instrument has newly and recently been developed, additional research is warranted to explore the several ways in which mindfulness may affect these outcomes, and, in turn, face-to-face social interactions [78].

### Study limitations and research implications

This study has some limitations that need to be discussed. The sample consisted of non-clinical adolescents from Lebanon, which may limit the generalizability of our conclusions to other populations and contexts. Future studies are required to test the psychometric characteristics of the original and short forms of the IMS, including in more diverse and larger Arabic-speaking samples from other Arab countries and communities worldwide. Moreover, additional research still needs to confirm the robustness of the scale in other populations, including in adult and clinical samples. A web-based method was employed to collect data, which may predispose us to data security issues and may limit the representativeness of our sample to the wider adolescent population as it has for example attracted more female respondents. Findings may also be subject to recall, response, self-selection and social desirability biases due to the self-report technique adopted. The long and short forms of the IMS were not tested independently in two separate samples. As no other measures of the interpersonal mindfulness construct are available to date in the Arabic language, we were not able to investigate convergent validity of the IMS in the context of the present study. Additionally, some psychometric properties were not examined in the context of the present study, such as test-retest reliability and cross-country invariance. Future longitudinal studies with different time-points are still needed to ensure that the factor structures of the short and long versions of the IMS remain stable over time, thus allowing changes in interpersonal mindfulness levels to be observed. As high quality evidence is available regarding the promise of incorporating mindfulness-based interventions in school settings for improving an array of psychological outcomes in youth [79], the two Arabic versions of the IMS may help implement, monitor, and assess the effectiveness of targeted and evidence-informed interventions aimed at promoting interpersonal relationships during the critical period of adolescence. This could also enable future experimental studies and research that solely focus on interpersonal mindfulness-based interventions to be conducted within Arab settings. Furthermore, establishing cross-gender invariance of the 27-item and 13-item IMS is important, as it might allow to develop gender-sensitive approaches in psychotherapy and educational settings - taking into account interpersonal experiences encountered by both males and females - in Arab societies, which are characterized by major gender inequalities in roles, rights, opportunities and responsibilities within communities and social institutions [80]. Finally, and pending future validation studies in clinical settings, the short Arabic version of the IMS can be particularly useful for Arab clinicians and researchers who often work under time and resource constraints, and will hopefully encourage future research in this little investigated topic in the Arab context.

## Conclusion

The present findings provide support for the good psychometric qualities of the Arabic translation of the IMS in both long and short forms, suggesting that these scales are suitable for use to measure interpersonal mindfulness in Arabic-speaking youth, at least in Lebanon. We expect that the IMS, in particular its shortest form, will prompt more systematic investigation of interpersonal mindfulness in the Arabic-speaking populations, especially with regard to enhancing healthy communications with others and building effective social relationships. More research is needed to confirm the factor structure of the Arabic 27-item and 13-item IMS, and their practical usefulness in the broader Arabic-speaking population.

## Data Availability

The datasets generated and/or analyzed during the current study are not publicly available due to the restrictions from the ethics committee but are available from the corresponding author on a reasonable request.
